# Migration Routes and Staging Areas of Trans-Saharan Turtle Doves Appraised from Light-Level Geolocators

**DOI:** 10.1371/journal.pone.0059396

**Published:** 2013-03-27

**Authors:** Cyril Eraud, Marcel Rivière, Hervé Lormée, James W. Fox, Jean-Jacques Ducamp, Jean-Marie Boutin

**Affiliations:** 1 Office National de la Chasse et de la Faune Sauvage, Villiers en Bois, France; 2 Maisonneuve, Saint Pierre d’Oléron, France; 3 Migrate Technology Ltd, Cambridge, United Kingdom; 4 Centre d’Etudes Biologiques de Chizé, CNRS-UPR 1934, Villiers en Bois, France; University of Milan, Italy

## Abstract

The identification of migration routes, wintering grounds and stopover sites are crucial issues for the understanding of the Palearctic-African bird migration system as well as for the development of relevant conservation strategies for trans-Saharan migrants. Using miniaturized light-level geolocators we report a comprehensive and detailed year round track of a granivorous trans-Saharan migrant, the European Turtle Dove (*Streptopelia turtur*). From five recovered loggers, our data provide new insights on migratory journeys and winter destinations of Turtle Doves originating from a breeding population in Western France. Data confirm that Turtle Doves wintered in West Africa. The main wintering area encompassed Western Mali, the Inner Delta Niger and the Malian/Mauritanian border. Some individuals also extended their wintering ranges over North Guinea, North-West of Burkina Faso and the Ivory-Coast. Our results reveal that all individuals did not spend the winter period at a single location; some of them experienced a clear eastward shift of several hundred kilometres. We also found evidence for a loop migration pattern, with a post-breeding migration flyway lying west of the spring route. Finally, we found that on their way back to breeding grounds Turtle Doves needed to refuel after crossing the Sahara desert. Contrary to previous suggestions, our data reveal that birds used stopover sites for several weeks, presumably in Morocco and North Algeria. This later finding is a crucial issue for future conservation strategies because environmental conditions on these staging areas might play a pivotal role in population dynamics of this declining species.

## Introduction

Populations of Afro-Palearctic migrant birds have suffered a sustained and severe decline for several decades [1]. Contrary to sedentary birds, understanding the causal mechanisms proves difficult because the underlying demographic processes may operate at different key stages in the life cycle of migrant species and across broad geographic ranges [2]. With regards to the reproductive phase, it has been shown that agricultural intensification could contribute to the decline in several trans-Saharan species, especially those breeding in farmland habitats (see [3]). However, comparative studies show evidence that population trends of long distance migrants wintering in dry and open areas in Africa were declining more significantly than those of short-distance migrants or residents [1], suggesting an influence of factors operating outside the breeding grounds.

It is well established that the environmental conditions experienced on the wintering grounds may significantly affect the population dynamics of trans-saharan migrants [4]–[9]. For instance, temporal variation in survival rates has been shown to be positively correlated with rainfall regimes [4]–[7] and proxies of food availability (i.e. NDVI, [9]). Relatedly, it is thought that food access and quality might be among the major proximate environmental factors shaping the survival of birds on their wintering quarters [4]. Besides this potent and direct impact on population dynamics, habitat quality and environmental conditions experienced by birds on their wintering grounds may also have delayed adverse effects on subsequent breeding success [10]. While such carry-over effects have been documented in few species [10]–[13], they are suspected to be more conspicuous in migratory birds than previously thought [14]. *En-route* conditions encountered by avian migrants may also have important effects on their population dynamics [15], through for example, weather-induced mortality [16], [17] and conditions experienced at stopover sites. While many trans-Saharan migrants are thought to overfly the desert by alternating migrating flights and stopover periods [18]–[21], they undertake a nonstop flight from a dietary perspective [21]. Hence, resting or fuelling conditions before crossing may play a crucial role in the success of migration journeys, on subsequent body condition and ultimately on individual fitness.

The identification of migration routes, major stopover and wintering regions and habitats are therefore crucial issues for understanding current population trends, predicting the consequences of changes in land use and developing appropriate conservation measures [3], [20], [22], [23]. However, we still lack fundamental data regarding these issues for the vast majority of trans-Saharan migratory species. This is particularly true for small- and medium-sized species for which direct survey methods such as satellite and GPS tracking remain inapplicable given the size and weight of devices. For these species, existing knowledge is mainly based on ringing schemes that usually provide sparse and biased data given the low and spatially highly heterogeneous band recovery rate in Africa [24]. However, the recent development of miniaturized light-level geolocators affords new perspectives in tracking small- and medium-sized species year-round and across broad geographical ranges [25]–[27]. Existing studies performed on trans-Saharan migrants have highlighted the valuable information that can be provided by these devices, even when only a few individuals are tracked [24], [27]–[32].

In the present study, we deployed light-level geolocators on trans-Saharan European Turtle Doves (*Streptopelia turtur turtur*). This species has declined over the whole of Western Europe [33]. This trend has been dramatic in some regions such as the UK, where breeding populations have declined by 70% since 1995 [34]. Agricultural intensification on the breeding grounds is suspected to be the main driver of this trend, through a shortening of the breeding season and a sharp decrease in productivity per pair [35], [36]. Recent works suggest that habitat quality and food resources on the sub-sahelian wintering grounds may have also significant impact on individual fitness [37]. This clearly exemplifies the relevance of demographic processes other than those operating on the breeding grounds and therefore, the need to gain a deep insight into the migration ecology of the species. In this context, our aim was to identify the migration routes of the species and its main wintering and stopover areas.

## Materials and Methods

### Ethics Statement

Turtle Doves were captured and handled in compliance with legal requirements (ONCFS licences N° 50, 3, and 16 delivered respectively to MR, JMB and CE). Birds were tagged with geolocators in compliance with the permission delivered by the national authority (Arrêté n° 2009–014, Préfecture de Paris). This authorisation permitted us to capture and equip Turtle Doves with data loggers across all of France in the period 2009–2014. Access to field sampling sites was allowed by the communal association of hunting (A.C.C.A. Saint-Pierre-d'Oléron).

### Study Site and Field Methods

The study area is situated along the French Atlantic coast, on Oléron Island (175 km^2^, 45.90°N, 1.30°W) where the Turtle Dove is a common breeder. The landscape is a mix of arable lands, marshes, small woody areas and villages. Adult doves were mist-netted or drop-trapped within the framework of a long-term research project designed to study the population dynamics of the species (see [37] for more details on capture sites and field methods). During the breeding seasons 2009 and 2010, miniaturized light-level geolocators were fitted to 33 and 31 (respectively) breeding adult Turtle Doves. Birds were tagged irrespective of their encounter histories (both newly captured and birds ringed on previous seasons). We only equipped Turtle Doves caught between late May and early July to avoid transient birds [37]. The next seasons (i.e. 2010 and 2011), we attempted to recapture birds using similar field methods.

### Geolocators and Light Data Processing

We used Mk14-S and Mk18 geolocators provided by the British Antarctic Survey. Turtle Doves roost and spend much time in trees and shrubs, with legs folded under the body. Loggers were therefore maintained on the back using an extendable wing-loop harness [38], with a light sensor mounted on a 2 cm stalk. Devices including harness weighed 2.5±0.07 g, corresponding to an average of 1.57±0.01% of birds’ body mass measured on the day of capture (mean: 160.06±1.42 g, *n* = 64). Some birds tagged with loggers (*n* = 16) were recaptured in subsequent days, allowing us to investigate the change in body condition following attachment. Their average body-mass change was +0.53±1.85%. This value did not differ significantly from the value measured in 29 control birds (+0.59±1.68%) that were not equipped with loggers (repeated measurements GLM including instrumentation as a fixed factor and the time (in days) elapsed between successive captures as a covariate; Instrumentation: *F*_1,42_ = 0.001, *P* = 0.977), suggesting that tagging did not alter body condition within this time scale (range: 4–86 days, overall mean: 22.8±2.9 days). In 2009, a total of 182 birds were captured and among them, 30 were recaptured during the subsequent season (i.e. 2010). In 2010, a total of 175 birds were captured and among them, 14 were recaptured in 2011. For each cohort, we found no evidence for a statistical difference in the recapture rate between tagged and control birds (cohort 2009∶ 15.2% *vs*. 16.8% respectively, χ_1_^2^ = 0.05, *P* = 0.82; cohort 2010∶ 9.7% *vs*. 7.6% respectively, χ_ 1_^2^ = 0.14, *P* = 0.70).

The Mk14-S and Mk18 geolocators measured light level every minute and recorded the maximum level at the end of every 10-minute or 5-minute period respectively (2009 and 2010 deployments respectively). These data were used to derive geographical positions using the threshold method [39]–[41]. Sunrise and sunset times were defined when the light level reached a threshold of 5 arbitrary units. We used BASTrak software (British Antarctic Survey 2009) to convert light data into location fixes following the BAS Geolocator Manual [42]. Turtle Doves usually inhabit woody and shaded environments and a high level of shading in the light data was evident. The integrity of each daily light curve was carefully inspected and sunrise/sunset events were rejected when necessary (discontinuous light curves, or those with an abrupt transition, … characteristic of inconsistent shading). Both noon and midnight data were included. We excluded latitude positions for approximately 21 days before and after autumnal and vernal equinox [39]–[41].

Of the 64 deployed loggers, 8 were retrieved in subsequent springs, with 5 containing full data. All geolocators received a static pre-deployment calibration in an open area at our lab during one week (WGS84∶ 46.15°N, 0.404°W, France). From this ‘rooftop’ calibration [43], the 5 retrieved loggers had almost identical light sensitivity. For a light level threshold of 5 units, the altitude of sun ranged from −4.95 to −5.50° and averaged –5.19±0.10°.

The value of −5.19° was not further used for determining locations of Turtle Doves. It is indeed unlikely that this light level threshold-sun elevation pair of values was representative of the habitats and weather conditions encountered by Turtles Doves on their breeding/wintering grounds and along their migration routes due to the more shaded vegetation in which the birds usually roost. For example, a radio-tracking survey of adults performed on Oléron Island revealed that birds roosted in small woods, hedges and/or shrubs with dense foliage (*unpublished data*). Unfortunately, the lack of knowledge of shading factors at dawn and dusk away from the deployment site makes accuracy very difficult to determine, and is the major factor limiting the accuracy of light level geolocation [43]. For our study, we adopted a strategy based on the use of two sun angles, each reflecting different parts of the deployment data. A first part included locations over Atlantic and Southern Europe zones and a second part covered locations over the African continent. To infer a sun angle for the first part of the deployment data, we took advantage of two birds recaptured in 2009 on their breeding sites, respectively 8 and 21 days after instrumentation. Using these live locations as a pre-deployment calibration period, we found that, for the same light level threshold, the solar elevation angle differed significantly from those calculated from rooftop calibration: −3.14° and −3.60° (mean: −3.37°) showing comparatively increased light attenuation. This difference is likely related to birds’ behaviour and the proximate environment; Turtle Doves are known to be active at half light and return back to their roosts either at dusk or half an hour before sunset [44].

Turtle Doves breeding in western France are thought to spend winter across Western African countries [45]. Given the more open habitat of the African region, it was thought that data gathered by a geolocator on a bird in this area would experience less light attenuation (less shading) than that encountered at the breeding site e.g birds roosting in less densely wooded areas and/or trees with less dense foliage (e.g. *Acacia sp.*, [46]). To infer a relevant sun angle for the African region, we followed recommendations of the manufacturer by proceeding to a static pre-deployment calibration within the presumed wintering range of the species [42]. To this aim, we deployed a Mk18 logger in Senegal (WGS84∶ 14.59°N/15.44°W) from 15 to 24 January 2011 in a typical tree (i.e. *Acacia sp.*) in an attempt to mimic roosting conditions. From these data, we calculated an average solar elevation of –4.66° (±0.17, *n* = 19). Average location data (± SE, *n* = 18 positions) was 14.53° N (±0.35) and 15.95°W (±0.07). Mean daily (± SE) error was calculated as 154±24 km during the calibration period, with longitude being more accurate (58±10 km) than latitude (137±23 km) (see also [39]–[40]). Hence, with our assumptions of roosting timing and habitat, we used a sun elevation angle of −3.37° to determine the geographical location of Turtle Doves during the breeding and migration phases over Atlantic and Southern Europe zones, and a sun angle of –4.66° for the wintering and migration phases over the African continent.

The robustness of our conclusions based on the use of these two distinct sun angles was evaluated by using different values derived from alternative hypotheses and approaches [31]. First, we performed supplementary analyses assuming that the critical sun angle equalled either the value inferred from the rooftop calibration (−5.19°) or the one inferred from live locations on breeding grounds (−3.37°). Second, we performed a “Hill-Ekstrom calibration” [43] based on data from the main staging periods in Africa (excluding active migration and periods of stopover). This analysis was performed using the R package GeoLight [47]. Results based on either the rooftop or the Hill-Ekstrom calibration (see Fig. S1 for values) conformed to our initial conclusions about migration routes and staging areas. The use of a sun angle of −3.37° produced unrealistic positions for most staging areas in Africa, either during the wintering or the spring migration period. Examination of the results revealed that staging sites covered unsuitable areas for the species (moist forest, ocean, etc) and that locations spread along an extended latitudinal gradient suggesting clearly a mismatch between light threshold value and the sun angle [48]. Full details are given as supporting material (Fig. S1).

### Data Analysis

Geographical positions of Turtle Doves were twice-smoothed [29], [49] and mapped using ArcMap 9.2 (ESRI) on a UTM31N projection. Migration routes, staging- and wintering areas were defined from positions along with their corresponding dates. We used the Home Range Tools 1.1 for ArcGIS [50] to separately derive Kernel density distribution maps for wintering and stopover areas during the spring migration. For each period, the average smoothing parameter across all individuals was used as the reference bandwidth. The grid size was set to 2 km [24] and density distribution maps included 50, 75, 90 and 95% percent of the kernel density. Given the intrinsic inaccuracy of geographical positions calculated from GLS data and the difference in accuracy between latitude and longitude, we emphasize that GLS data do not meet the basic assumptions of the kernel method. Hence, the resulting kernel density contours should not be interpreted as home-ranges but only viewed as synthetic maps summarizing the distribution of estimated locations. Similarly, because kernel contours encompass the inaccuracy of data, their extent should not be viewed as a firm evidence that birds were not stationary at specific sites. In the Turtle Dove, post-breeding migration phase usually overlap autumnal equinox. During this period, accurate latitudinal data were not available. Hence, we used only longitude data to assess both migration dates and corridors [25], [28]. When latitudinal data were available, migration schedule and flyways were approximated directly from the positioning data [24], [29]. Data can be accessed at the following link: doi:10.5061/dryad.bv24t.

## Results

### Autumn Migration

**Figure 1:**
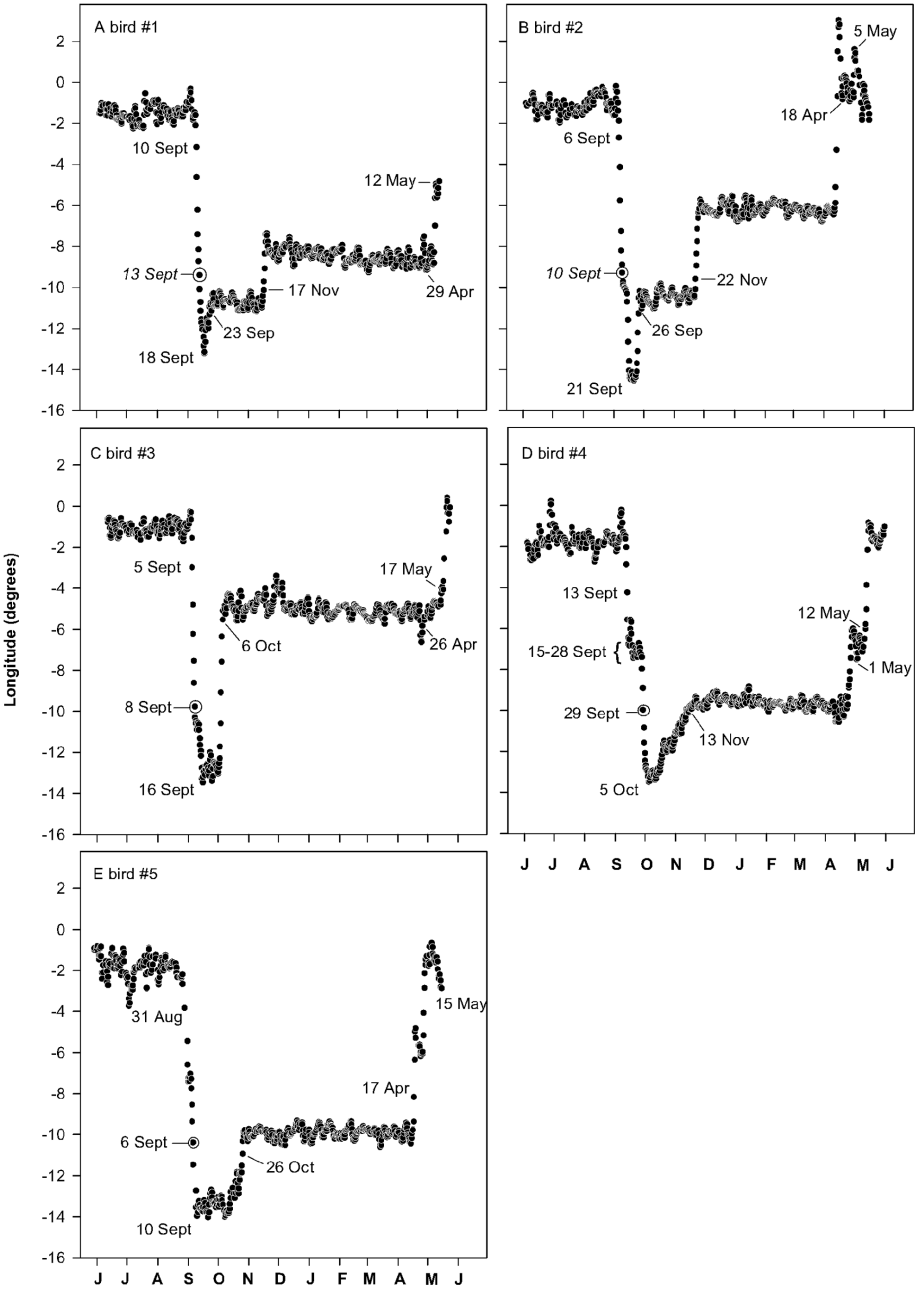
A year of longitude variations for Turtle Doves fitted with geolocators in 2009 (A–C) and 2010 (D–E). Dots correspond to both noon and midnight locations in WGS84 degrees (twice smoothed). Key dates are indicated (see §Results, Tab. 1). The circled points correspond to the dates when longitude data indicated that birds were located either off the coasts of Portugal or over Moroccan/Mauritanian territories (see also Fig. 2 depicting migration pathways from that date). Data can be accessed at: doi:10.5061/dryad.bv24t.

**Figure 2:**
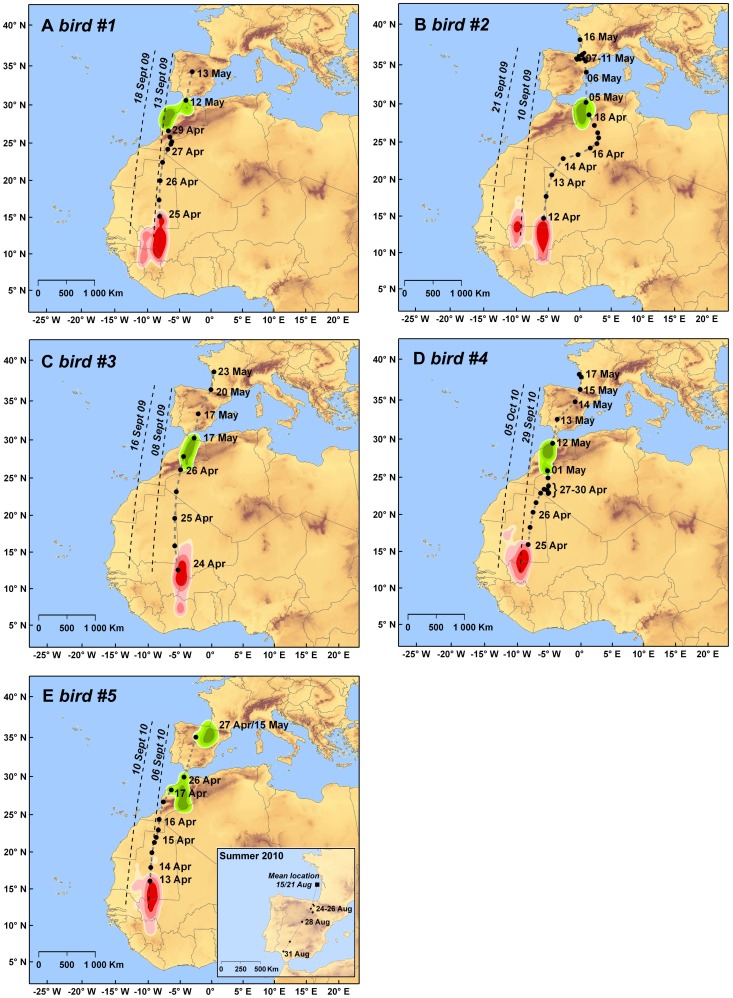
Estimated migration routes, stopover- and wintering areas of Turtles Doves with key dates. Density contours reflect 50, 75, 90 and 95% kernel density. Dashed lines depict the migration corridor used by the birds in autumn, when longitude data indicated that they were clearly off the coasts of Portugal or over Moroccan or Mauritanian territories (See also Fig. 1). Wintering grounds are coloured red and are based on all locations (twice a day) outside the autumnal equinox period (from 12 Oct) and until departure date in the following spring. Staging areas are coloured green. Note that kernel contours do not illustrate home ranges because the inaccuracy of data is embedded in their calculation. The spring migration routes derived from (twice smoothed) positioning data are shown along with the corresponding dates for illustrative purposes. Note that single positions (black dots) and migration paths (dash lines) are approximated on the basis of geolocation data with low accuracy. The migration route for bird#5 that started its autumnal migration earlier is framed in box. Note that the location on 31 August was at the extreme bounds of the exclusion period (i.e. 31 Aug/12Oct.). Bird IDs are similar to Fig. 1 and Tab. 1. The loggers of birds #1 and 5 ceased collecting data en-route. Data can be accessed at: doi:10.5061/dryad.bv24t.

Four individuals undertook their post-breeding migration around autumnal equinox, when migration dates and routes could be only assessed on the basis of longitude data. Significant changes in longitudinal data in early September (mean = 6 September ±2.2 days) show evidence that Turtles Doves were engaged in active migration at that time (Fig. 1). These early signs of movement were followed by a rapid westward progression. Longitude values attained by mid-September (6–13 Sept., Fig. 1) indicated that from that date, the doves used a migration corridor located off the coasts of Portugal and/or over Moroccan and Mauritanian territories (Fig. 2). Bird#4 departed from this general pattern, entering the corridor at a later date (i.e. 29 Sept) after a plausible stop-over from 15 to 28 September (Fig. 1D). Bird#5 started migration earlier, giving latitudinal data for a short period. In this case, we could show that this bird was engaged in an active autumnal migration before that was detected by a significant change in longitude by 31 August (Fig. 1E). At that date the bird was located near the Strait of Gibraltar following a short-term stopover in Northern Spain (Fig. 2E).

Overall, birds took on average 2 weeks (13.2±2.5 days) to reach their most western locations. Afterwards, longitude values remained stable for several consecutive weeks (but see Fig. 1A) suggesting a plausible use of staging areas. As we were unable to determine latitude, we cannot tell whether birds stopped north from Sahara over Moroccan/Mauritanian territories, or alternatively south from Sahara over northern Senegal until late September/early October (Fig. 1B–C, E). At that time, birds rapidly moved eastward (Fig. 1A–C). In 2009, the eastward transition ended between 23 September and 6 October. Corresponding longitude values matched those we attributed to wintering areas, suggesting that birds tagged in 2009 reached their wintering quarters by late September/early October, after a 3–4 week migration (mean = 21.3±5.3 days, Fig. 1A–C). Birds tagged in 2010 followed a similar flyway but with a slightly different timing, remaining on their westernmost locations until mid-October and then reaching their main wintering grounds later (26 Oct and 13 Nov, Fig. 1D,E).

### Wintering Period

Minimum and maximum latitudes of individual core areas (i.e. 50% kernels) ranged between 10.7°N and 18.1°N, whereas longitudes ranged between −4.1°W and −10.9°W. Two of the Turtle Doves tagged in 2009 spent most part of the winter in Mali, with their 50% core areas centred over the Inner Niger Delta (Fig. 2B, C). The third one (i.e #1), spent most part of the winter in south western Mali (Fig. 2A). Interestingly, we found evidence for a clear eastward shift of wintering quarters for two birds. These changes occurred rapidly in one or two days in the second half of November (Fig. 1A, B). Before its shift, bird#1 was located about 150 km further west until mid-November, with locations extending southwards in Guinea (Fig. 2A). Until 22 November, bird #2 was located 400 km westwards on both sides of the Malian/Mauritanian border, (Fig. 2B). A roughly similar area was used as the main wintering ground by the two Turtle doves tagged in 2010 (Fig. 2D,E).

### Spring Migration

Spring migration took place outside the vernal equinox, allowing departure dates to be inferred from shifts in latitudinal data. Departure from the wintering quarters occurred from 12 to 25 April (mean = 19 April ±3 days). On the whole, individuals crossed the Sahara Desert straight through Mauritania or close to Malian border, during a migratory journey of 2.5–6.5 days (Fig. 2). Bird #2 took a slightly different route from northern Mali, by crossing Algeria via the Adrar province (Fig. 2B). The travelled distances ranged between 1 560 and 2 350 km, with a corresponding flight speed ranging between 240 and 812 km.day^−1^ (Table 1).

**Table 1:** Detail of the migration schedule of the five Turtle doves equipped with geolocators in 2009 (birds #1–3) and 2010 (birds #4–5).

		Autumn migration				Spring migration			
Bird	Data	Significant shift in longitude^a^	Most western location^a^	Main wintering grounds^b^	Eastwards shift	Departure fromwintering grounds	No. of traveldays	Distance^c^	Speed (km.day^−1^)^d^
**#1**	Partial	10 Sept	18 Sept	**Mauritania/Mali**/Guinea	17 Nov	25 Apr	4	1 568 km	392.0
**#2**	Good	6 Sept	21 Sept	**Mauritania/Mali**	22 Nov	12 Apr	6	2 350 km	391.7
**#3**	Good	5 Sept	16 Sept	**Mali**/Burkina Faso/Ivory Coast		24 Apr	2.5	2 031 km	812.4
**#4**	Good	13 sept	05 Oct	**Mauritania/Mali/**		25 Apr	6.5	1 561 km	240.1
**#5**	Partial	31 Aug	10 Sept	**Mauritania/Mali**/Guinea		13 Apr	4	1 682 km	420.5

a Values are derived from Fig. 1.

b Locations of the core wintering areas (50% Kernels) are in bold.

c Cumulate distance between successive fixes.

d Calculated as distance/No of travel days.

Longitude values (Fig. 1) and positioning data (Fig. 2) indicated that on their way back to breeding grounds, Turtle Doves followed a loop migration pattern, taking a more eastern flyway than during autumn migration. Importantly, results also revealed that birds did not directly return back to Europe after crossing the Sahara but stopped over in Morocco or in the border region with Algeria (#2, Fig. 2B). Only one individual (i.e. #4), used a short-term stopover at the extreme west of Algeria before reaching its main stopover area (Fig. 2D). Birds left their stopover areas between 26 April and 17 May (mean = 8 May ±3.5 days), after spending 14±2 days on average (Table 2). Almost all Turtle Doves crossed the Mediterranean Sea in the vicinity of the Straight of Gibraltar.

**Table 2:** Detail of the migration schedule of the five Turtle doves equipped with geolocators (continued).

Bird	Arrival on stopover	No. of staging days	Departure from stopover	Arrival on breeding grounds	Date of recapture
**#1**	29 Apr	13	12 May	?	26 May
**#2**	18 Apr	17	05 May	16 May	18 May
**#3**	26 Apr	20.5	17 May	23 May	26 May
**#4**	01 May	10.5	12 May	17 May	31 May
**#5**	17 Apr^a^	9	26 Apr		
	27 Apr^b^	18	?	?	23 June

a Stopover located in North Africa.

b Stopover located in Spain.

Two loggers ceased collecting data when birds were crossing Spain (#1, 5). Hence, it was not possible to determine the full course of their ascent to their breeding grounds. From the three remaining loggers and using the closest locations around capture sites as evidence for arrival on Oléron Island (Table 2), we determined that the final migratory journeys lasted around 7.3±1.9 days. The accumulation of locations in Northern Spain for bird#2 suggests that this bird staged before crossing the Pyrenees Mountains (Fig. 2B). A staging area was also used in Spain by bird #5 for more than two weeks before its logger stopped collecting data (Fig. 2D).

Based on birds #2, 3 and 4, we calculated that the spring migration from wintering grounds (including stopover duration) lasted 28.3±3.5 days (Tables 1 & 2).

## Discussion

Overall, our results shed new light on major features of the migration pattern of Eurasian Turtle Doves originating from Western Europe. The amount of information gained by only 5 loggers clearly outweighs the sum of current knowledge gained by more than a decade of intensive ringing effort performed on this species in France. Of the 1150 adults and pulli ringed since 2001 on Oleron Island by our team, none were recovered in Africa. All ringed birds were recovered in autumn either in France or over the Iberian Peninsula (Fig. S2) thus limiting our capability to draw an essential picture of the entire annual migratory cycle.

### Localisation of Wintering Grounds

On the basis of the migration schedule derived from geolocators with complete data (i.e. #2, 3, 4), the average annual time allocated to autumn and spring migration would represent 18±2%, whereas about half the year (51±3%) would be spent on the wintering grounds (see also [24], [32]).

Turtle Dove populations breeding in Western Europe are thought to spend winter in sub-Sahelian regions of West Africa where food resources (weed seeds and split grains) are abundant, water is available and roosting conditions are ensured (woody cover) [45], [51], [52]. Unsurprisingly, all tracked individuals spent winter in West Africa and in regions where, plausibly, environmental characteristics met the requirements of the species (see Fig. S3, S4, S5). On the basis of compiled data on ring recoveries [45] and various published observational data [53]–[55] we initially expected the winter range of our birds to be within Senegambia. Our results suggest that this was not the case since all core areas were distributed more easterly. Two individuals spent most part of the winter in the Mali’s Inner Delta Niger, therefore corroborating the importance of this area suggested by several authors [45], [54] as well as by our own observations (Fig. S5). Two other individuals wintered on both side of the Malian/Mauritanian border, illustrating that individuals originating from the same breeding population may use different wintering quarters (see also [24]).

Our results also shed new light on the potential southern limit of the wintering range of individuals originating from Western Europe. It appears that the wintering ranges of some individuals could encompass northern Guinea, north-west of Burkina Faso and the edge of Ivory Coast. This southern limit was previously suspected [54], [55] but never confirmed, owing to the difficulty to discriminate among individuals of the nominate subspecies *S.t. turtur* from those belonging to the subspecies *S.t. arenicola* breeding in North Africa. However, given the inaccuracy embedded in GLS data (see also Fig. S1), further studies are needed to confirm this result.

We also found evidence that some individuals did not spent the whole course of the wintering period at a single location but could operate a clear eastward shift of several hundred kilometers (Fig. 2B, see also [32]). It is unlikely these shifts may have been caused by disturbance occurring on roosting sites, for which the species is sensitive [51], because these shifts took place in the same direction, roughly at the same period and concerned two individuals that were initially located on distinct regions. A more plausible hypothesis could be that these shifts corresponded to birds tracking water and/or food resources made available by the maturing and harvesting of cereal crops (millet, sorghum, rice).

### Evidence for a Loop Migration

The use of different migration routes in autumn and spring is a migration strategy of several Afro-Palearctic migrant species [56]. Some studies have clearly found evidence for such a loop migration in Hoopoes [24] and Red-backed Shrike *Lanius collurio* [31] fitted with geolocators. Our results suggest a similar but less marked pattern, with an autumnal migration route lying west of the spring route. One bird (# 2) has clearly made a large loop, returning to North Africa across Algeria via the province of Adrar. We cannot exclude that this migration path was unusual and possibly taken in response to adverse conditions encountered during the migratory journey, such as headwinds and dust storms.

In spring, the vast majority of birds returned to the Iberian Peninsula across the Mediterranean sea through or in the vicinity of the Strait of Gibraltar. This result suggests that the loop migration of Doves would concern only the the Sahara part of the migration. The uncertainty caused by the period of equinox did not allow us to integrate latitude data to determine the precise route taken by migratory birds leaving the European continent. A similar pattern of longitude data would be produced by birds reaching the Moroccan coast either by sea from the coasts of Portugal or by cutting through Spain and the Strait of Gibraltar (Fig. 1). Data provided by bird # 5, which had started its migration before the equinox, support the latter hypothesis although recoveries of birds ringed on Oléron Island suggest that all birds would not converge to Gibraltar (Fig. S2). The autumnal migration corridor depicted on Fig. 2 might therefore correspond to a westward movement operating along the Atlantic coasts of Morocco as suggested by observational data [57], [58].

Possible explanations for the distinct migration routes of the Turtle doves include seasonal differences in habitat quality and/or a response to seasonal meteorological conditions, especially wind conditions [31]. However, it remains to be confirmed if this migration strategy is predominant or variable among individuals, populations or across years.

### Turtle Doves Make Long Stopovers

Prior to crossing the Sahara in spring, Turtle Doves are known to increase their fat reserves south of the desert’s edge [45], [59]. Existing data suggest a huge weigh gain [53] and it is thought that this fuel load would enable Turtle Doves to cross the Sahara and to reach the southern Europe in a non-stop flight [45]. However, our results show clearly that this was not the case. During spring migration, some birds crossed the Sahara in 2.5 to 4 days (#1, 3, 5). For these fastest individuals, the covered distance ranged between 1 500 and 2 000 km (Tab. 1), corresponding to an approximate average flight speed of 392–812 km.day^−1^. Under the hypothesis that birds have made a non-stop flight, the estimated flight speed is far below the flight velocity recorded in other columbid species (43–56 km.h^−1^ for *Zenaida macroura* [60], *Columba oenas* [61] respectively). Assuming a non stop flight and a mean flight speed of 49 km.h^−1^, most individuals would have reached their stopover areas in less than two days. While we acknowledge that the determination of departure and arrival times is fairly imprecise, these results suggest that most individuals probably used short stops when crossing the Sahara [21]. But more importantly, our results show that all birds would stop over for several weeks before crossing the Mediterranean Sea. Similarly to the observed distribution of birds during the wintering phase, our results indicate that birds from the same population did not converge to a single stopover site, but used different areas presumably distributed over Morocco and Western Algeria. The Turtle doves spent on average 56±7% of the spring migration period at these stopover sites (Birds#2,3,4; range: 48–71%, Tab. 2). In early spring, adult Turtle doves are supposed to have completed the moult of their flight-feathers (wing-and tail-feathers) [53], so it is likely that the birds used this time to restore their body condition before reaching their breeding grounds further north. In support of this hypothesis, our results show that all stopover areas included farmlands devoted to cereal cropping (Fig. S3). In addition, an anecdote mentioned by Morel [53] reported significant damages to cereal crops by Turtle doves during the spring passage in Morocco. Although the key role of North-Western African territories as stopover region in spring was previously suspected [62], to our knowledge this is the first time that their pivotal role during the course of the spring migration would be established. We also found that one individual (bird #5) operated a long stopover in Spain, succeeding a short stop in Morocco. This would suggest that individuals might adopt different migration strategies, plausibly in response to various factors including fuel accumulation or physiological state after crossing the Sahara.

Unlike the spring migration, our works provide only limited insights on the strategy adopted by birds during fall migration. Our results showed that the birds stabilized their longitude at the extreme west for several consecutive days, suggesting the use of stopover sites before reaching the wintering grounds. However, their locations remain to be elucidated. Where the transition between breeding and wintering grounds occurs during a period of equinox, light level threshold analysis of geolocator data is insufficient to establish this.

### Conservation Implications

The speed by which some individuals have crossed the Sahara desert demonstrates the magnitude of the effort sustained by birds. As emphasized by Schmaljohann et al. [21], it is likely that birds do not refuel *en route* but have to perform a non-stop flight “*in the sense of nutritional terms*”. This suggests that the pre-migratory fattening process prior to spring migration might play a crucial role in determining the success and the costs of subsequent migratory journeys [20]. The species has been previously shown as highly sensitive to human-caused disturbance on its resting sites [50]. Accordingly, the avoidance of any disturbance at both roosting and feeding sites during this period should be encouraged in order to maximize the fattening process [45].

Perhaps a more relevant demonstration of the cost paid by Turtle Doves when crossing the Sahara Desert is the duration of subsequent stopovers, lasting up to 3 weeks. This suggests clearly that birds need to restore their body condition before continuing further north. In a previous study, we found that a significant part of the variance in annual survival was explained by environmental conditions encountered by birds on their wintering grounds [37]. The current study suggests that those encountered by birds on their stopover areas might also play a crucial role in driving population dynamics. The key ecological requirements of the species on these staging regions are poorly known and should therefore receive increased attention.

## Supporting Information

Figure S1 **Estimated migration routes, stopover- and wintering areas of Turtles Doves according to alternative values of sun angle.**
(PDF)

Figure S2 **Adult Turtle Doves ringed in Oléron Island (Western France) during the breeding season and recovered in foreign countries.**
(PDF)

Figure S3 **Harvested area of cereals as the proportion of each grid cell.**
(PDF)

Figure S4 **Tree cover as the proportion of each grid cell.**
(PDF)

Figure S5 **Known locations of Eurasian Turtle Doves in Western Africa during autumn and winter.**
(PDF)
