# Supplementary material for: Migration Routes and Staging Areas of Trans-Saharan Turtle Doves Appraised from Light-Level Geolocators
Source: PLoS One. 2013 Mar 27;8(3):e59396. doi: 10.1371/journal.pone.0059396 (PMC3609750; doi:10.1371/journal.pone.0059396)
Supplement: Figure S1 — Estimated migration routes, stopover- and wintering areas of Turtles Doves according to alternative values of sun angle. (PDF) [file pone.0059396.s001.pdf]

Figure S1. Estimated migration routes, stopover- and wintering areas of Turtles Doves according to alternative values of sun angle.

Density contours reflect 50, 75, 90 and 95% kernel density. Wintering grounds are coloured red and are based on all locations (two per day) outside the autumnal equinox period (from 12 Oct) and until departure date in the following spring. Staging areas are coloured green. Black dots indicate spring migration routes derived from positioning data.

Alternative sun angles included i) the value calculated from the rooftop calibration performed at our lab (-5.19°), ii) the value calculated from live locations on breeding grounds (-3.37°) and iii) values calculated from a Hill-Ekstrom calibration approach based on data corresponding to the main staging period in Africa (excluding active migration and periods of stopover).

A thoroughly examination of these results tends to support the validity of our initial approach based on the use of the sun angles -3.37° and -4.66° for different parts of the deployment data. When using a sun angle of -3.37° several results do not conform to existing knowledge about the ecology of the species. First, wintering areas are clearly located south of the sub-Saharan region, over dense and moist forested areas. Locations are distributed over the whole Ivory Coast, Guinea, Sierra Leone, Liberia and into the Gulf of Guinea. Second, several stopover areas used during the spring migration fell either into the Atlantic Ocean or the Mediterranean Sea. In addition, the density of locations during the wintering period (summarized by kernel contours) spread along a much more extended latitudinal gradient compared to results achieved from an angle of -4.66°. The dates related to the corresponding locations were those that were closer to the period of equinoxes. This suggests that a sun angle of -3.37° was inappropriate for assessing wintering grounds and stopovers. Indeed, according to Ekstrom (2004 Mem. Natl Inst. Polar Res., 58), i) the latitude error increases with increasing mismatch between light threshold value and sun angle and ii) this error is magnified for locations near the equinoxes.

With a sun angle of -5.19°, differences with the initial approach are slighter. Overall, wintering areas are located north from locations estimated from a sun angle of -4.66°. However, this discrepancy does not affect the overall conclusions about the location of wintering grounds and stopovers, migration paths and schedule. We stress however that the wintering areas of some birds (i.e. #2, 4 & 5) would be located over the arid zone of the sahelian band which is somewhat unexpected for a granivorous species that needs both large amount of seeds and water. In addition, for bird #4, two set of locations stand out clearly from the core of the wintering area, both north and south. The corresponding dates prove to be the closest to the period of equinoxes. Therefore, it is likely that this result reflects once again a mismatch between light threshold value and the value of -5.19°. Hence, these elements suggests that a sun angle of -5.19° was less appropriate than a sun angle of -4.66° to track Turtle Doves in Africa. It appears also that -5.19° was also inappropriate for the European part of the deployment data since locations failed to predict arrival on breeding sites (i.e. Oléron Island, see Birds #2, 3, 4).

Overall, results based on the Hill-Ekstrom approach conform to our initial main conclusion about migration routes and staging areas over the African continent.

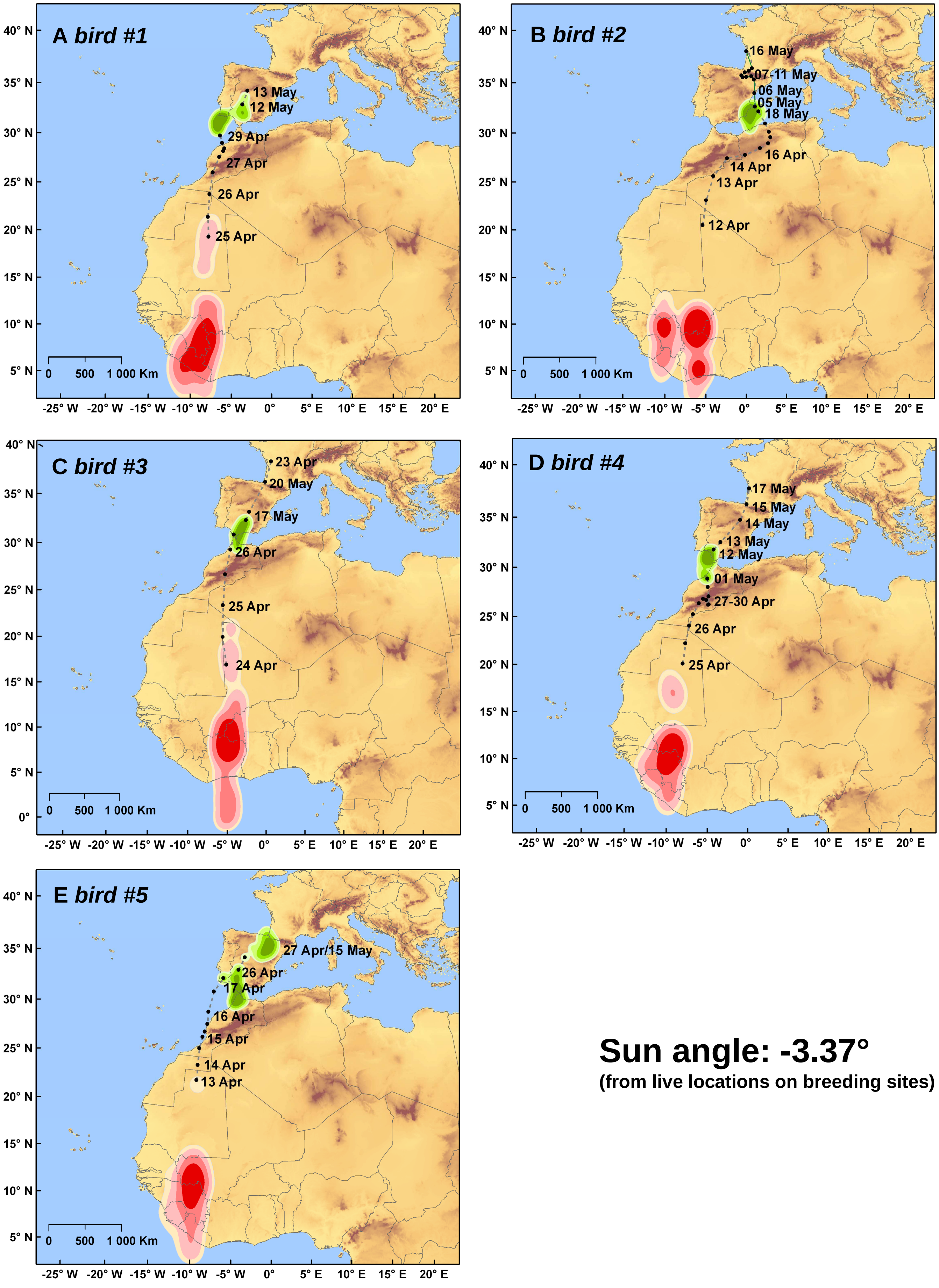

A bird #1
13 May
12 May
29 Apr
27 Apr
26 Apr
25 Apr
B bird #2
16 May
07-11 May
06 May
05 May
18 May
16 Apr
14 Apr
13 Apr
12 Apr
C bird #3
23 Apr
20 May
17 May
26 Apr
25 Apr
24 Apr
D bird #4
17 May
15 May
14 May
13 May
12 May
01 May
27-30 Apr
26 Apr
25 Apr
E bird #5
27 Apr/15 May
26 Apr
17 Apr
16 Apr
15 Apr
14 Apr
13 Apr
0 500 1 000 Km
40° N
35° N
30° N
25° N
20° N
15° N
10° N
5° N
0°
-25° W -20° W -15° W -10° W -5° W 0° 5° E 10° E 15° E 20° E
Sun angle: -3.37°
(from live locations on breeding sites)

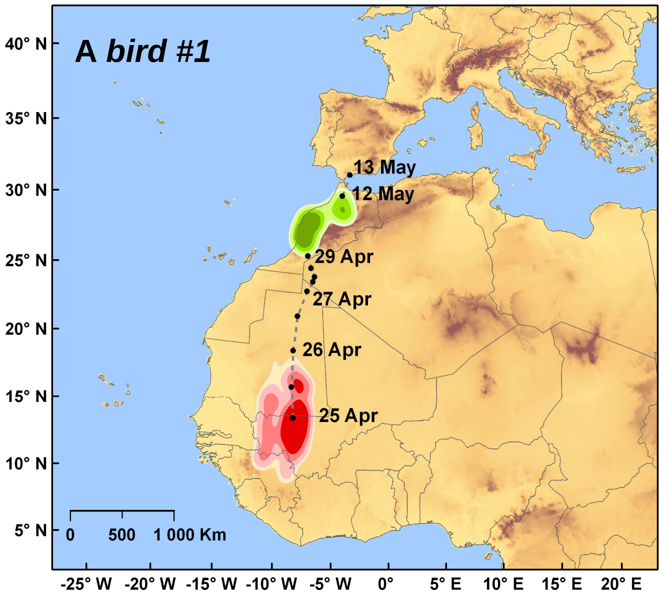

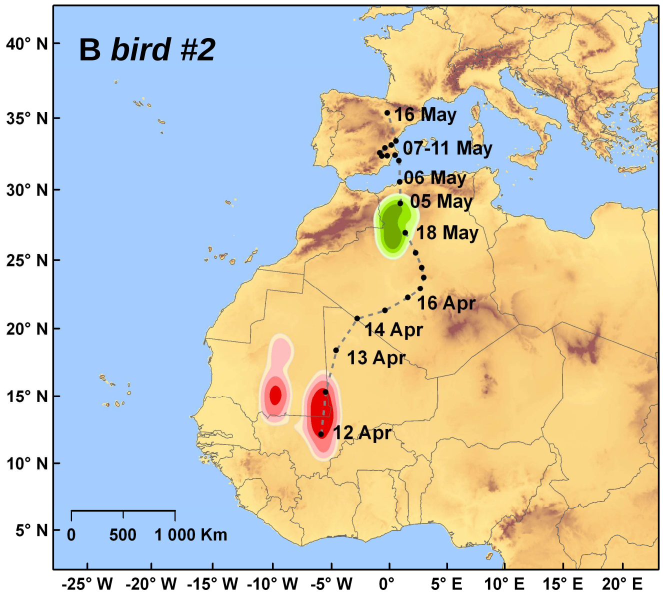

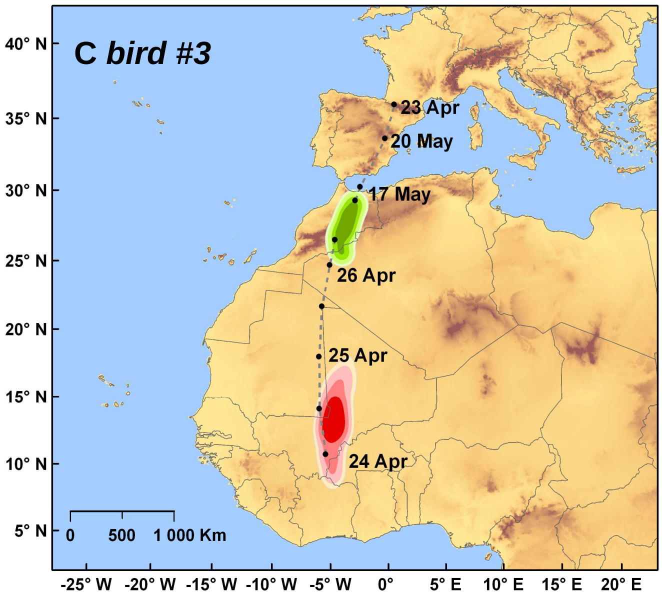

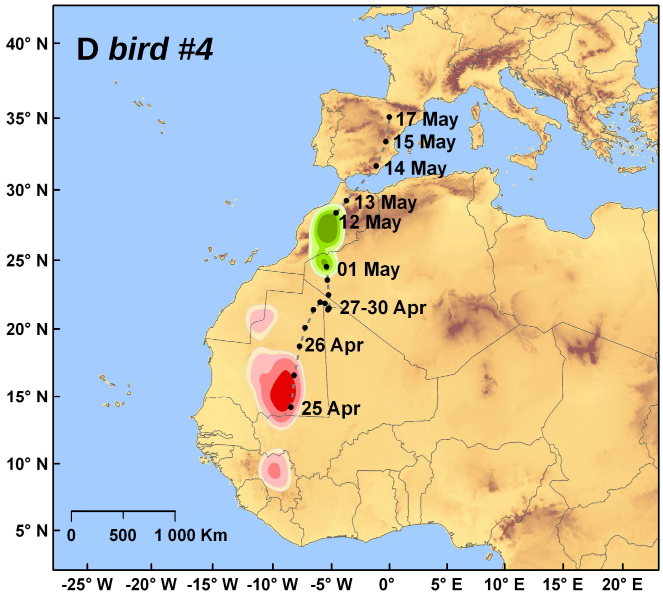

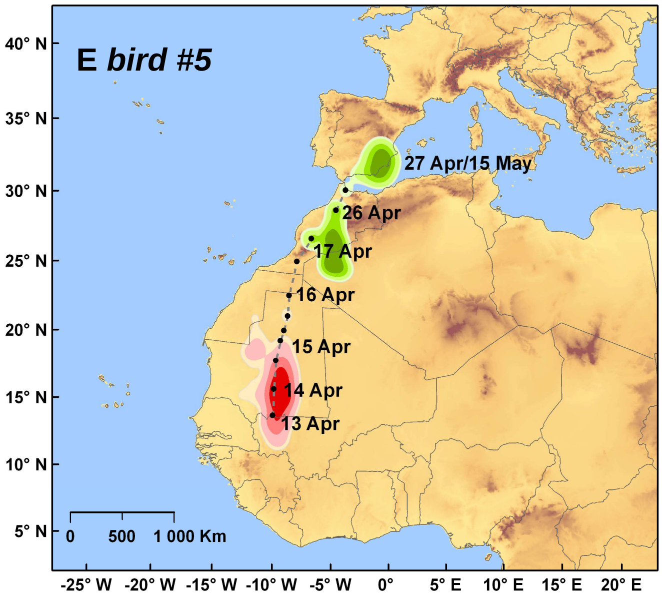

Sun angle: -5.19°

(from rooftop calibration at Lab)

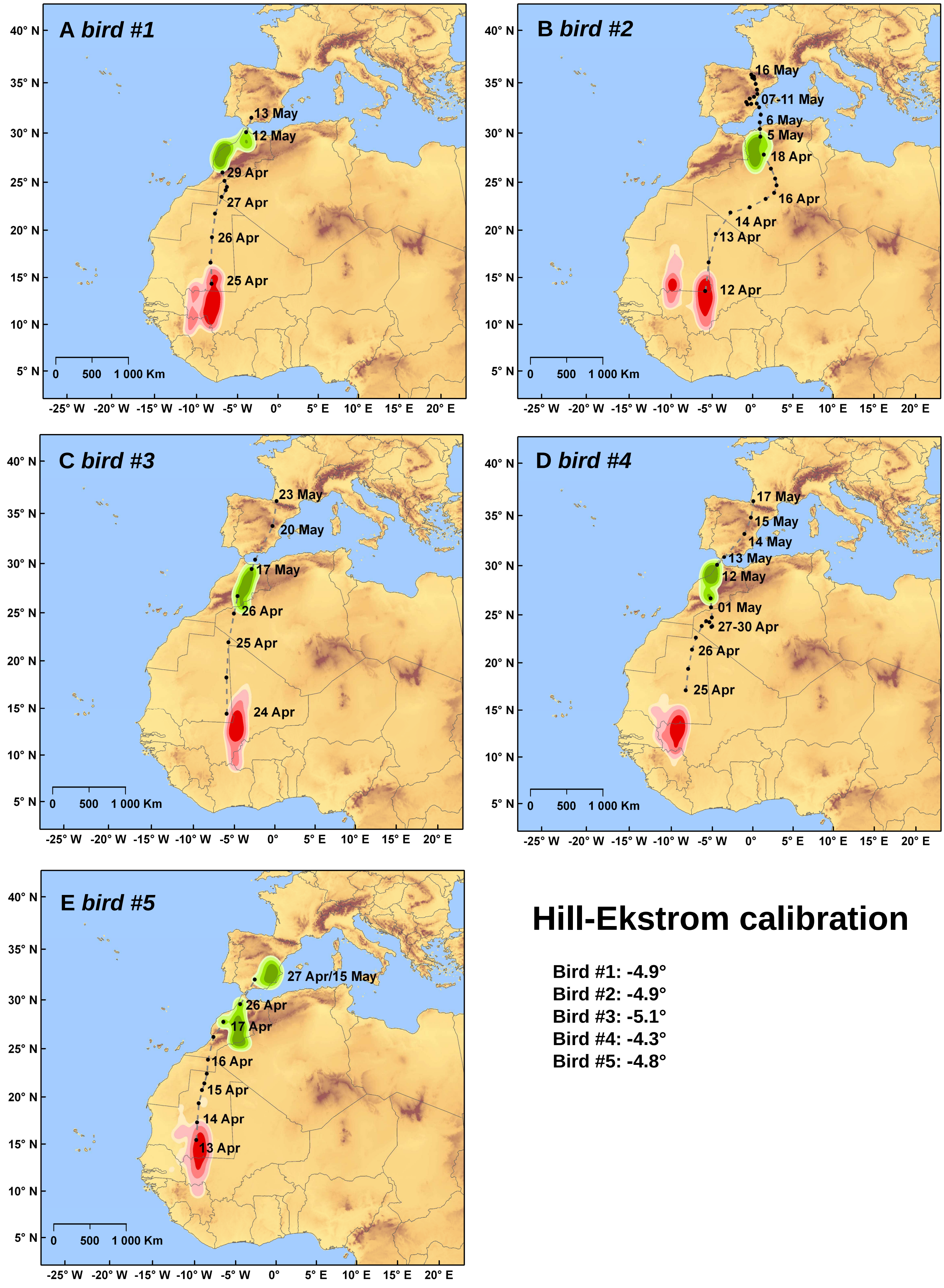
A bird #1
13 May
12 May
29 Apr
27 Apr
26 Apr
25 Apr
B bird #2
16 May
07-11 May
6 May
5 May
18 Apr
16 Apr
14 Apr
13 Apr
12 Apr
C bird #3
23 May
20 May
17 May
26 Apr
25 Apr
24 Apr
D bird #4
17 May
15 May
14 May
13 May
12 May
01 May
27-30 Apr
26 Apr
25 Apr
E bird #5
27 Apr/15 May
26 Apr
17 Apr
16 Apr
15 Apr
14 Apr
13 Apr
0 500 1 000 Km
40° N
35° N
30° N
25° N
20° N
15° N
10° N
5° N
-25° W
-20° W
-15° W
-10° W
-5° W
0°
5° E
10° E
15° E
20° E
Hill-Ekstrom calibration
Bird #1: -4.9°
Bird #2: -4.9°
Bird #3: -5.1°
Bird #4: -4.3°
Bird #5: -4.8°
