# Supplementary material for: Migration Routes and Staging Areas of Trans-Saharan Turtle Doves Appraised from Light-Level Geolocators
Source: PLoS One. 2013 Mar 27;8(3):e59396. doi: 10.1371/journal.pone.0059396 (PMC3609750; doi:10.1371/journal.pone.0059396)

Figure S2. Adult Turtle Doves ringed in Oléron Island (Western France) during the breeding season and recovered in foreign countries. Recovery dates are the following: 1) 15 Sept 1999, 2) 02 Sept 2000, 3) 05 Sept 2002, 4) 12 Sept 2002, 5) 24 Sept 2004, 6) 30 Aug 200, 7) 09 Sept 2007.

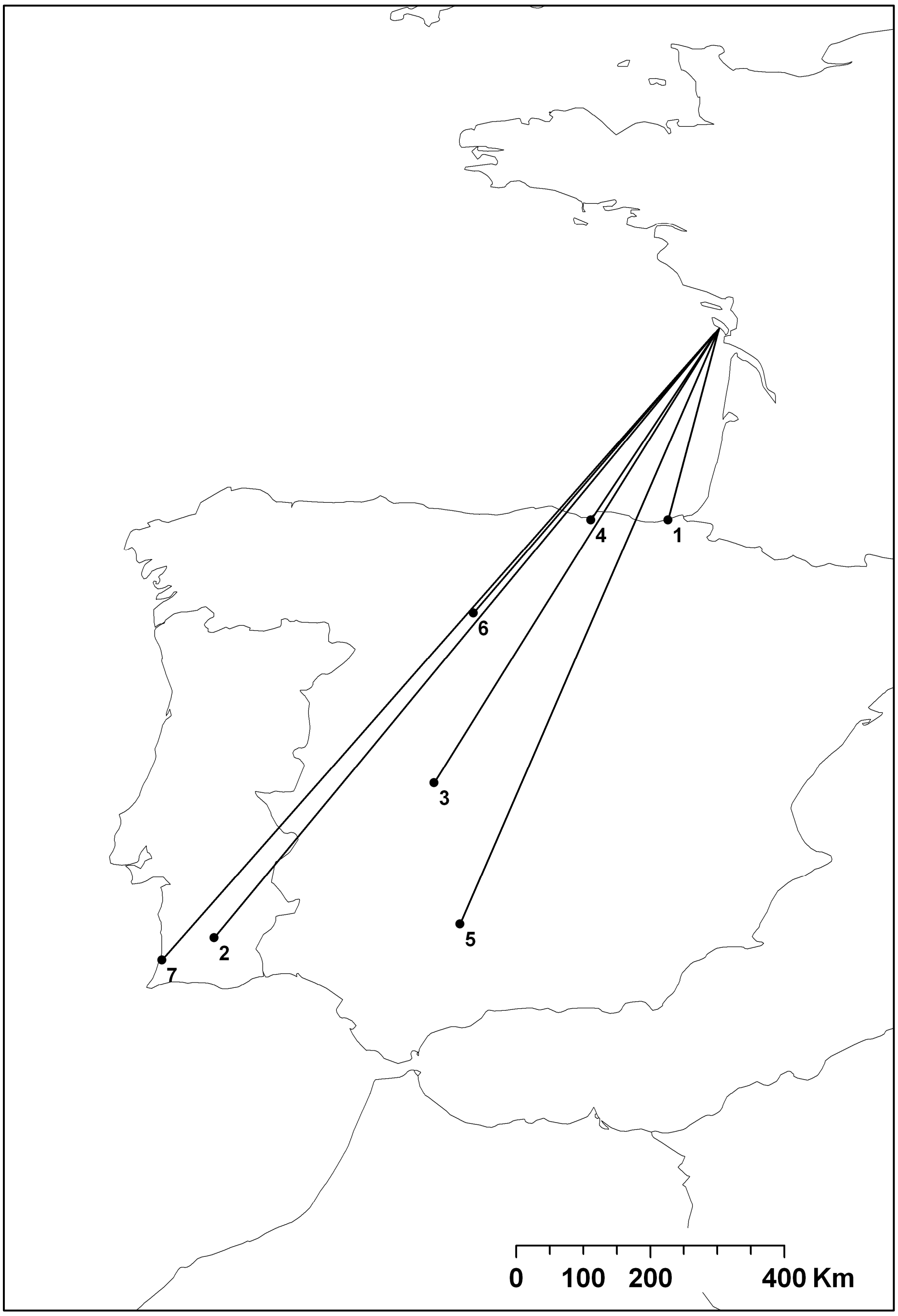

Supplement: Figure S2 — Adult Turtle Doves ringed in Oléron Island (Western France) during the breeding season and recovered in foreign countries. (PDF) [file pone.0059396.s002.pdf]
