# Supplementary material for: Migration Routes and Staging Areas of Trans-Saharan Turtle Doves Appraised from Light-Level Geolocators
Source: PLoS One. 2013 Mar 27;8(3):e59396. doi: 10.1371/journal.pone.0059396 (PMC3609750; doi:10.1371/journal.pone.0059396)

Figure S3.Harvested area of cereals as the proportion of each grid cell.

Source: Monfreda C, Ramankutty N, Foley JA (2008) Farming the planet: 2. Geographic distribution of crop areas, yields, physiological types, and net primary production in the year 2000. Global Biogeochem Cycles, 22: GB1022, doi:10.1029/2007GB002947. The map was drawn from data downloaded at: http://www.geog.mcgill.ca/~nramankutty/Datasets/Datasets.html.

75% kernel contours are shown with one colour assigned to each bird.

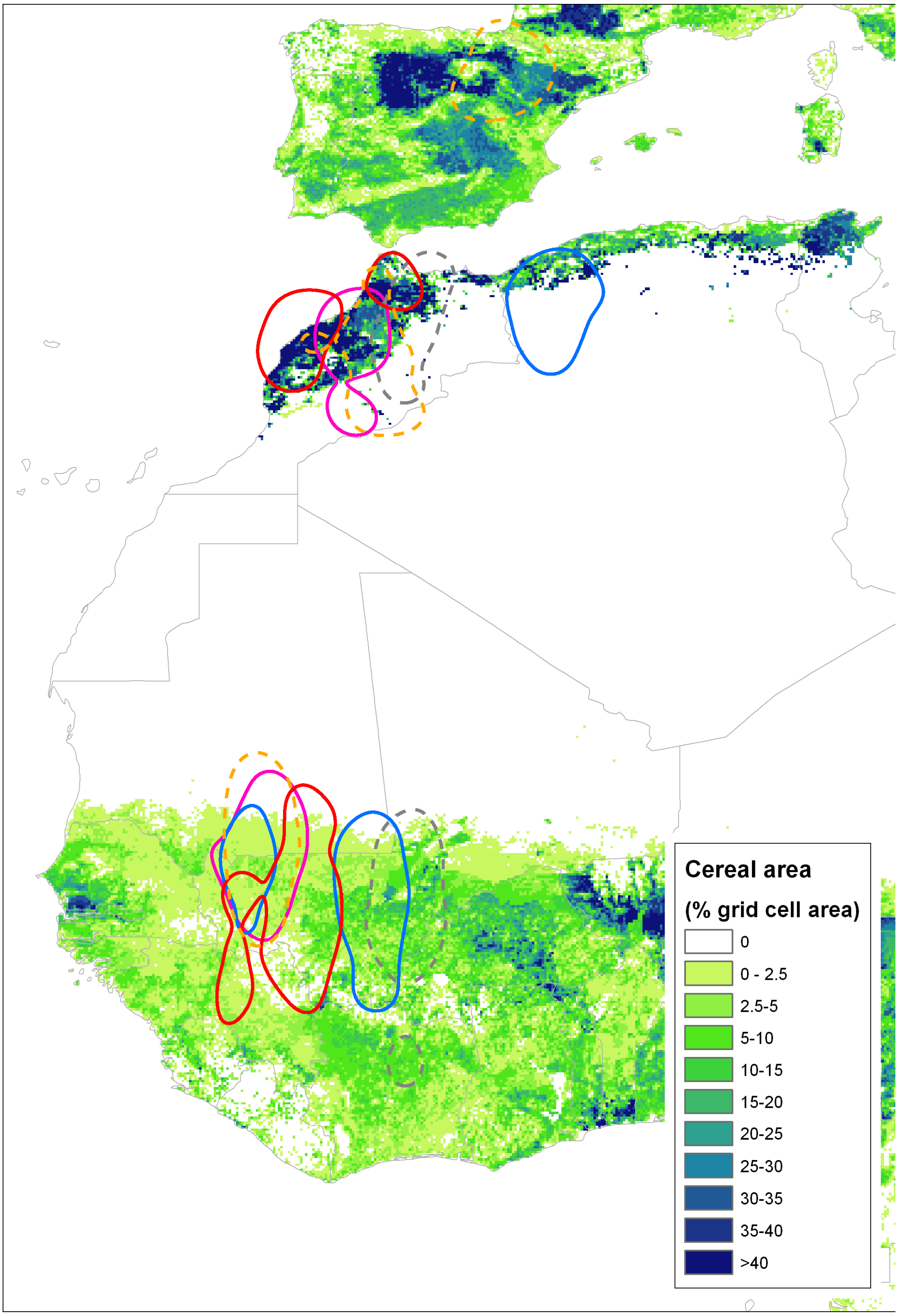

Supplement: Figure S3 — Harvested area of cereals as the proportion of each grid cell. (PDF) [file pone.0059396.s003.pdf]
