# Supplementary material for: Migration Routes and Staging Areas of Trans-Saharan Turtle Doves Appraised from Light-Level Geolocators
Source: PLoS One. 2013 Mar 27;8(3):e59396. doi: 10.1371/journal.pone.0059396 (PMC3609750; doi:10.1371/journal.pone.0059396)

Figure S4. Tree cover as the proportion of each grid cell.

Sources: Derries R, Hansen M, Townshend JRG, Janetos AC, Loveland TR (2000), 1 Kilometer Tree Cover Continuous Fields, 1.0, Department of Geography, University of Maryland, College Park, Maryland, 1992-1993; Defries RS, Hansen MC, Townshend JRG, Janetos AC, Loveland TR (2000) A new global 1-km dataset of percentage of tree cover derived from remote sensing. Global Change Biol 6: 247-254. The map was drawn from data downloaded at: Global Land Cover Facility, http://www.landcover.org./.

75% kernel contours are shown with one colour assigned to each bird.

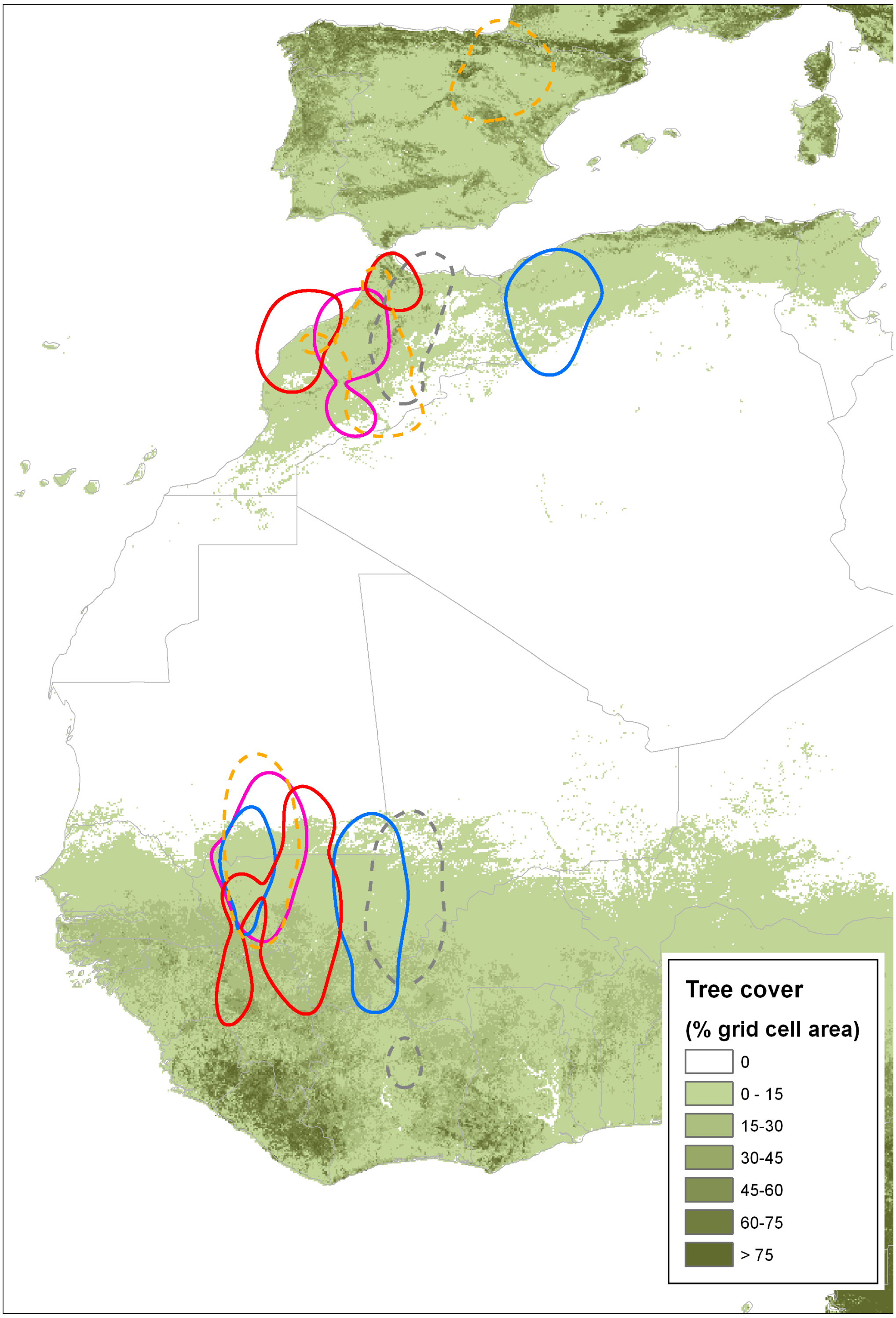

Supplement: Figure S4 — Tree cover as the proportion of each grid cell. (PDF) [file pone.0059396.s004.pdf]
