# Supplementary material for: Migration Routes and Staging Areas of Trans-Saharan Turtle Doves Appraised from Light-Level Geolocators
Source: PLoS One. 2013 Mar 27;8(3):e59396. doi: 10.1371/journal.pone.0059396 (PMC3609750; doi:10.1371/journal.pone.0059396)
Supplement: Figure S5 — Known locations of Eurasian Turtle Doves in Western Africa during autumn and winter. (PDF) [file pone.0059396.s005.pdf]

Figure S5. Known locations of Eurasian Turtle Doves in Western Africa during autumn and winter.

Data are synthesized from the following sources: **a**) Morel G, Morel M (1988) Alauda 56, **b**) Morel G (1987*)* Malimbus 9, **c**) Girard O – ONCFS *unpublished data*, **d**) Boutin J.-M., Girard O – ONCFS *unpublished data*, **e**) Jarry G (1994) Statut et Biologie de la Tourterelle des Bois. Rapport interne CRBPO, Paris, **f**) Jarry G, Baillon F (1991) Hivernage de la Tourterelle des Bois (*Streptopelia turtur*) au Sénégal : Étude d'une Population dans la Région de Nianing. Rapport interne CRBPO, Paris. When available, the date and number of birds seen (*in italics*) are given. Observations from d and e were recorded during field surveys dedicated to waterbirds censuses. Note that all observations do not distinguish between the two subspecies (i.e. *S. t. turtur* and *S. t. arenicola*). For some observations, coordinates were derived from location names cited in the original text. Inland waters are shown in blue (data downloaded at: http://www.diva-gis.org).

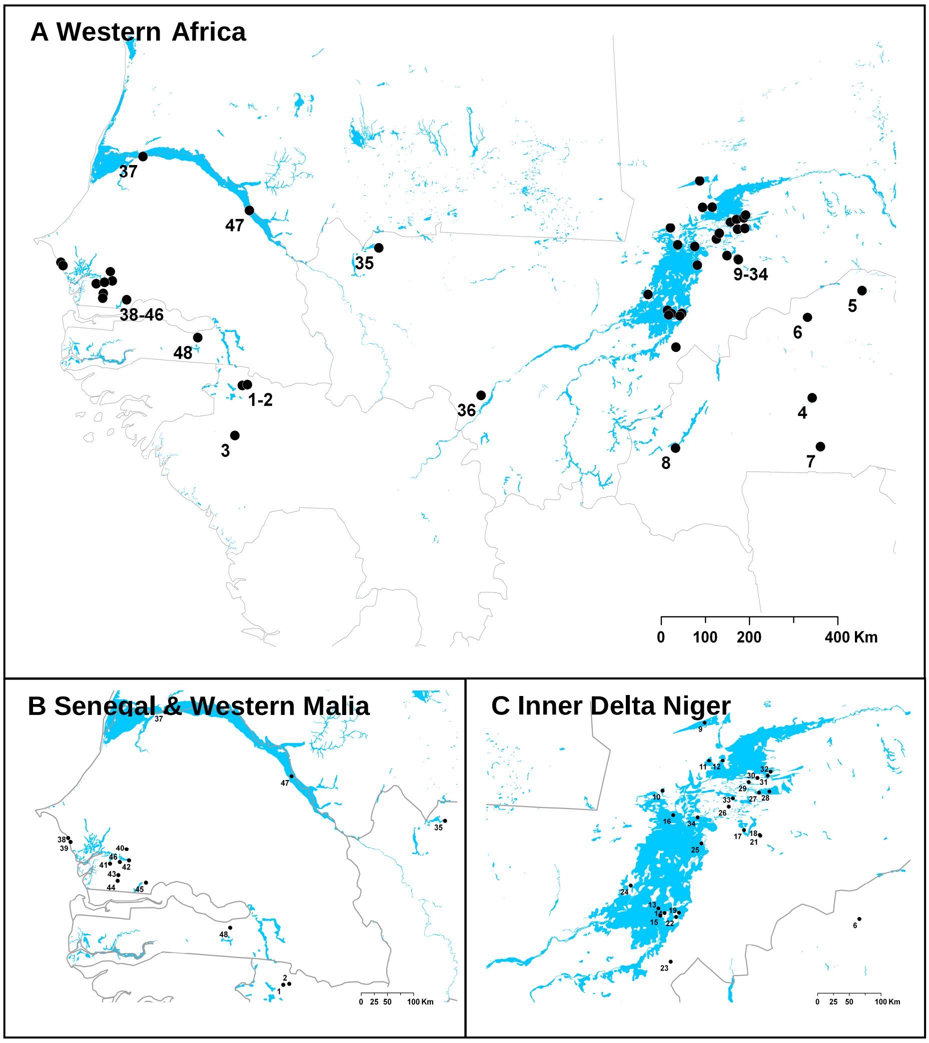

Guinea . – **1**) 19 Jan 1987 (*small groups*) [a], **2**) 21 Jan 1987 [a], **3**) 26 Jan 1987 [a],

Burkina Faso. – **4**) 8 Nov-6 May (*small groups of 10*) [b], **5**) Oct 1986 (*100 000*) [b], **6**) Apr 1983 (*1000*) [b], **7**) Jan 1983 [b], **8**) Jan 1983 [b],

Mali. – **9**) autumn/winter[b], **10**) autumn/winter [b], **11**) autumn/winter [b], **12**) autumn/winter [b], **13**) 18 Jan 2000 (*100*)[c], **14**) 19 Jan 2000 (*200*) [c], **15**) 19 Jan 2000 (*150*) [c], **16**) 19 Jan 2000 (*1 500*) [c], **17**) 23 Jan 2000 (*3 000*) [c], **18**) 23 Jan 2000 (*500*) [c], **19-20**) 13 Jan 2001 (*500*) [c], **21**) 14 Jan 2001 (*150*) [c], **22**) 14 Jan 2001 (*500*) [c], **23**) 14 Jan 2001 (*700*) [c], **24**) 10 Jan 2008 (*20*)[d], **25**) 11 Jan 2008 (*50*)[d], **26**) 13 Jan 2008 (*120*) [d], **27**) 13 Jan 2008 (*150*) [d], **28**) 13 Jan 2008 (*2 500*) [d], **29**) 13 Jan 2008 (*30*) [d], **30**) 13 Jan 2008 (*500*) [d], **31**) 13 Jan 2008 (*200*) [d], **32**) 13 Jan 2008 (*300*) [d], **33**) 14 Jan 2008 (*400*) [d], **34**) 15 Jan 2008 (*150*) [d], **35**) Mar 1975 (*100 000*) [b], **36**) 10 Jan 2007 (*2 000*) [d],

Senegal. – 37) [e], **38**) 27 Feb 1992 (*20 000*) [e], **39**) 23 Feb 1990 (*50 000*) [f], **40**) 1992 [e], **41**) Jan 1994 [e], **42**) 07 Mar 1994 (*55 000*) [e], **43**) [e], **44**) (*3 000*) [e], **45**) 25 Mar 1994 (*566 000*) [e], **46**) [e], **47**) [e] , **48**) [e].
